# Rapidly progressive pulmonary hypertension and right ventricular failure in a heart and kidney transplant recipient

**DOI:** 10.1002/ccr3.6631

**Published:** 2022-12-05

**Authors:** Matthew Deicke, Laith Alhuneafat, Omar Obaid, Aderonke Adeniyi, Amresh Raina, Hayah Kassis‐George

**Affiliations:** ^1^ Department of Internal Medicine, Allegheny Health Network Allegheny General Hospital Pittsburgh Pennsylvania USA; ^2^ Department of Cardiology St. Mary Medical Center Hobart Indiana USA; ^3^ Alice Hyde Medical Center University of Vermont Health Network Malone New York USA; ^4^ Section of Advanced Heart Failure and Pulmonary Hypertension Cardiovascular Institute, Allegheny Health Network, Allegheny General Hospital Pittsburgh Pennsylvania USA

**Keywords:** adenocarcinoma, cadaveric renal transplantation, immunosuppression, lymphangitic carcinomatosis, orthotopic heart transplantation, pulmonary hypertension, right ventricular failure, solid‐organ transplantation

## Abstract

A 54‐year‐old man status post heart and kidney transplant presented with dyspnea. Imaging was consistent with lymphangitic carcinomatosis (LC), in the setting of biopsy proven adenocarcinoma. He developed pulmonary hypertension (PH) and died of right ventricular failure (RVF) 3 weeks later. Acute PH with radiographic features of LC in a high‐risk patient warrants expedited malignancy investigation.

## Graphical abstract

Lymphangitic carcinomatosis is an under‐recognized manifestation of metastatic malignancy—resulting in fatal pulmonary hypertension and right ventricular failure—thus necessitating consistent and aggressive cancer screening in high‐risk patients, such as those who are immunocompromised and have a higher likelihood of the development of cancer.

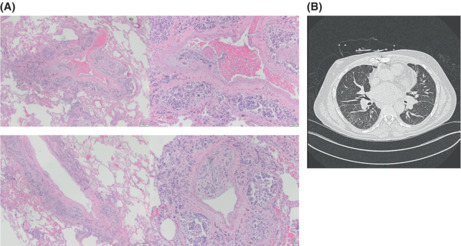

## 1 INTRODUCTION

Lymphangitic carcinomatosis is a common but under‐recognized cancer manifestation characterized by diffuse invasion of the lymphatic system by malignant tumor cells. We report a dramatic presentation of LC in a patient post double‐organ transplantation resulting in rapidly progressive pulmonary hypertension and subsequent RV failure. We describe the initial presentation and the accelerated clinical deterioration of this patient, emphasizing the importance for both a high index of suspicion and prompt investigation when acute RV failure develops in the post‐transplant patient population.

## 2 CASE HISTORY

A 54‐year‐old African American man status post orthotopic heart and cadaveric renal transplantation, systemic hypertension, obesity, and obstructive sleep apnea initially presents with signs of upper respiratory infection and diarrhea. Multiple other family members had similar symptoms, but while they improved, dyspnea and functional decline worsened for our patient. Prior to his presentation, he had been in a good state of health with normal allograft systolic function, normal invasive hemodynamics and no evidence of rejection on routine surveillance endomyocardial biopsies.

Initial vitals included an oxygen saturation of 86% on room air, heart rate of 101, blood pressure of 126/82 mmHg, and a body mass index of 38 kg/m^2^. Physical examination was notable for bibasilar rales and bilateral 2+ pitting lower extremity edema.

## 3 INVESTIGATION AND TREATMENT

Laboratory data included a white blood cell count of 6.76 k/mcl, hemoglobin 18.1 g/dl, hematocrit 54%, platelets 151 k/mcl, proBNP 534 pg/ml, creatinine 1.45 mg/dl, and electrolytes within normal limits. Patient was continued on home dose of high‐dose mycophenolate due to his elevated risk of rejection for an African American man, as well as tacrolimus for which the his blood level was checked and was found to be within the goal therapeutic range.

Initial chest radiography revealed pulmonary vascular congestion and pulmonary edema. Transthoracic echocardiogram (TTE) confirmed normal size and function of the left ventricle; however, reduced right ventricular systolic function based on tricuspid annular systolic plane excursion (TAPSE) 14 mm (normal ≥ 17 mm) with normal right ventricular size was also seen, similar to a TTE performed several months prior and consistent with postoperative findings in the setting of treated OSA and normal pulmonary pressures.

Right heart catheterization (RHC) was performed revealing an elevated mean pulmonary arterial pressure of 38 mmHg, a pulmonary vascular resistance of 10.4 woods units (WU) and a Fick cardiac index of 1.8 alongside a pulmonary arterial occlusive pressure (PAOP) of 2 mmHg. The patient had a negative fluid challenge (rise in pulmonary arterial occlusive pressure ≤ 15 mm Hg) suggesting no evidence of occult postcapillary pulmonary hypertension. Table 1. Right ventricular endomyocardial biopsy was also performed at the time of the catheterization and confirmed the absence of rejection or opportunistic infection.

**TABLE 1:** Right heart catheterization hemodynamics

Procedure Date	RA	RV s/d, RVEDP	PA s/d	PA m	PCWP	FCI	PVR
8/5	3	53/3, 4	53/27	38	2	1.8	829
8/19	15	86/12, 19	88/46	61	16	1.1	1626

Abbreviations: EDP, end diastolic pressure; FCI, FICK cardiac inde; PA, pulmonary artery; PAOP, pulmonary artery occlusion pressure; PVR, pulmonary vascular resistance; RA, right atrium; RV, right ventricle.

**FIGURE 1:**
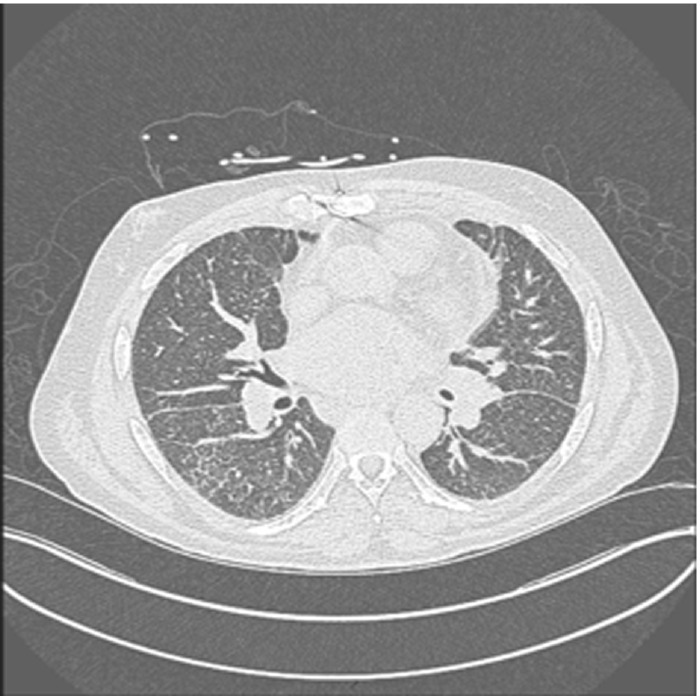
Initial CT scan revealing hilar adenopathy, LUL consolidation versus mass and chronic nodular septal thickening

Following the RHC, investigation for etiology of precapillary pulmonary arterial hypertension ensued. A ventilation perfusion scan was unremarkable, and CT imaging of the chest revealed a left upper lobe consolidation, hilar adenopathy, and nodular septal thickening Figure 1. A bronchoscopy was pursued postimaging with a cytopathology from a bronchoalveolar lavage negative for viral infections and silver stains negative for *Pneumocystis jirovecii*. Respiratory cells were benign with macrophage evidence suggesting old hemorrhage. Fine needle aspiration of two adjacent lymph nodes was performed with histology consistent with metastatic adenocarcinoma exhibiting papillary features.

Malignancy work up included ultrasound of the head and neck showing a mildly enlarged but homogenous thyroid gland, a 3 mm left sided nodule or cyst; however, no predominant solid mass or micro calcifications to suggest thyroid adenocarcinoma. Thyroid function tests were within normal limits as well.

Four days later, the patient developed multiple episodes of post‐tussive syncope and worsening hypoxia, with an increase oxygen requirement from 4 L/min to 15 L/min.

Repeat TTE revealed worsening moderate‐to‐severe right ventricular dysfunction with apical sparing and a TAPSE of 8 mm down from 14 mm seen previously. Right ventricular area fractional change was also reduced at 12% (normal ≥ 35%), and tricuspid annulus tissue pulsed Doppler was 8 cm/s (normal > 10 cm/s). Agitated saline bubble study ruled out interatrial right to left shunting and left ventricular systolic function was again found to be normal.

CT imaging was repeated and excluded an acute pulmonary embolus, however, revealed interval worsening of the previously demonstrated interstitial process, now with ill‐defined tree‐in‐bud nodules and worsening lymphadenopathy. A repeat RHC was performed as well, now showing pulmonary pressures approaching systemic pressures. Table 1.

Salvage radiation to the left upper lobe was considered as the patient was rapidly deteriorating. Infectious disease was involved due to the possibility and concern for a rapidly progressive opportunistic infection. Broad‐spectrum antibiotics, antifungals, and antiviral medications were initiated with cultures collected, all eventually returning negative.

The patient's clinical status continued to deteriorate. Pulmonary pressures as measured via Swan Ganz catheter now exceeded systemic pressures despite inhaled nitric oxide. Palliative care were involved, and ultimately the decision was made to make the patient comfortable. He then expired 24 days following his initial presentation.

**FIGURE 2:**
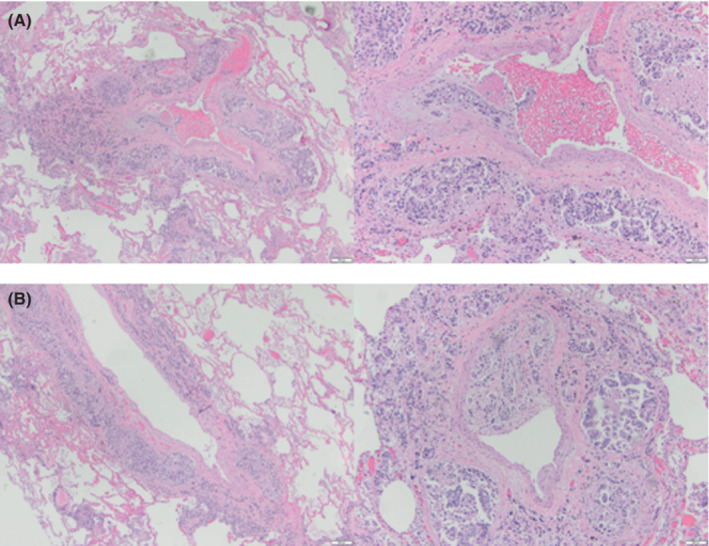
Histopathology. Extensive angiolymphatic invasion by tumor cells. Diffuse infiltration precludes accurate identification of vascular structures. (A) (left sided images) Low resolution (10×) and (B) (right sided images) High resolution (20×)

On autopsy, histopathologic diagnosis of pulmonary lymphangitic carcinomatosis was made. There were no other significant findings on autopsy of the thorax and abdomen/pelvis Figure 2. There were numerous hilar lymph nodes suspicious for metastatic disease though a primary cancer remained unknown.

## 4 DISCUSSION

We present a case of a heart and kidney transplant recipient with a rapid clinical decline after being found to have a left upper lobe mass with biopsy‐proven adenocarcinoma, lymphangitic spread resulting in profound hypoxemia, severe pulmonary hypertension, and expiring secondary to right ventricular failure. Lymphangitic carcinomatosis is not rare but it is often under‐recognized and associated with a very poor prognosis.

Lymphangitic carcinomatosis is associated more commonly with certain rapidly progressive malignancies, but it has been described in numerous neoplastic processes and is rarely found in isolation without a nearby satellite lesion or lymphadenopathy.^1^, ^2^, ^3^ While it can be mistaken on imaging for any disease process involving the lymphatic system, such as pulmonary edema, prompt identification of lymphangitic carcinomatosis is paramount, as it tends to follow an aggressive course as an end‐stage manifestation of malignancy resulting in death within 3 months of diagnosis.^3^ It is thought to be under‐recognized, and often not detected until postmortem via autopsy as seen in this case.

Our patient demonstrated no clinical signs or symptoms of malignancy until the time of presentation when CT imaging demonstrated adenopathy and a left upper lobe mass. Patients with LC typically present with nonspecific shortness of breath, and thus the clinician must have a high index of suspicion in order to expedite diagnosis and cancer therapy to help slow the disease progression as much as possible.^1^, ^2^

Local inflammatory responses result in edema characterized by bronchovascular wall thickening and alveolar septal thickening. X‐ray findings typically include thickened bronchovascular markings and Kerley B lines indicative of fluid overload and pulmonary edema as was seen in our patient.^4^ CT findings typically show interlobular septal thickening and local growth of metastasized tumor may also be detected as nodules on CT imaging.^3^, ^5^ Our patient had septal thickening and one ill‐defined pulmonary nodule.

Young patients who undergo solid‐organ transplantation (SOT) have decreased life expectancy when compared to the general population due to post‐transplantation complications such as acute or chronic graft rejection, infection, and death from malignancy.^6^, ^7^ There is a nearly 9% risk of developing a noncutaneous nonlymphomatous malignancy at 5 years, and for all malignancies, there is a 30% incidence rate at 10 years of post‐SOT.^8^, ^9^ Therefore, early detection and screening in post‐transplant patients is imperative to improve long‐term mortality. Our patient's risk factors for post‐transplant malignancy included being a male recipient, his age, and prior tobacco use history, as well as a high dose of mycophenolate because of his higher risk of rejection based on African American race.^8^, ^10^

The 2010 International Society of Heart and Lung Transplantation (ISHLT) has a guideline on the long‐term care of transplant recipients, including recommendations on the approach to malignancy.^11^ It is recommended that transplant patients undergo the same cancer screening as the general population. Under the 2013 and 2021 United States Preventative Services Task Force (USPSTF) current recommendations, certain high‐risk individuals should undergo lung cancer screening with low‐dose computed tomography (LDCT) scans annually (grade B recommendation).^12^

Rosenbaum et al.^13^ conducted a retrospective study in 2005 investigating the incidence of lung cancer in heart transplant recipients with a positive smoking history and the modalities utilized to detect these occult malignancies. CT scan was able to detect cancer four times more often when compared to chest X‐ray and six times more likely to identify stage I disease. By implementing annual screening for high‐risk patients with LDCT, cancer can be detected at earlier stages, therefore making patients candidates for resection, curative treatment and improving overall survival. This patient did not have any CT scans post‐transplantation until the time of his presentation, though CXR done throughout the entire post‐transplant period did not reveal a solid mass.

While the adenocarcinoma identified in our patient had papillary features not commonly seen with a lung cancer, along with lymphangitic involvement, precapillary pulmonary hypertension, and an otherwise unremarkable evaluation with regard to the origin of cancer, does suggest a primary pulmonary etiology. Our patient's precipitous decline may have been avoided or delayed, had his malignancy been identified with CT imaging in time to undergo effective chemo or radiation therapy—and with a reduction in immunosuppression—that is prior to the development of lymphangitic spread.

## 5 CONCLUSION

Solid‐organ recipients have a higher likelihood of developing cancer when compared to the general population. These malignancies are often quite aggressive, severe, and poorly responsive to treatment given the immunocompromised state of this patient population. This case emphasizes that early detection via screening is paramount if the goal is to pursue curative therapies or to delay mortality in the post‐SOT population.

## AUTHOR CONTRIBUTIONS

Matthew Deicke MD and Laith Alhuneafat MD were involved in writing review and editing. Omar Obaid DO and Aderonke Adeniyi MD were involved in visualization, data curation, and writing original draft. Amresh Raina MD was involved in data curation, writing review and editing, supervision. Hayah Kassis‐George MD was involved in visualization, data curation, writing original draft, writing review and editing, supervision, and project administration.

## FUNDING INFORMATION

The authors have no financial disclosures.

## CONFLICT OF INTEREST

The authors have no conflicts of interest or financial disclosures.

## CONSENT

Written informed consent was obtained from the patient's wife to publish this report in accordance with the journal's patient consent policy.

## Data Availability

Patient‐specific identifying information was omitted, and no patient information was altered or falsified in an attempt to maintain anonymity. Consent was obtained from the patient's next‐of‐kin for permission to publish.
